# CGP7930 - An allosteric modulator of GABA_B_Rs, GABA_A_Rs and inwardly-rectifying potassium channels

**DOI:** 10.1016/j.neuropharm.2023.109644

**Published:** 2023-11-01

**Authors:** Saad B. Hannan, Reka Penzinger, Ginte Mickute, Trevor G. Smart

**Affiliations:** Department of Neuroscience, Physiology and Pharmacology, University College London, Gower Street, London, WC1E 6BT, UK

**Keywords:** GABA, GABA_A_ receptors, GABA_B_ receptors, Neuronal inhibition, CGP7930, Potassium channels, Positive allosteric modulator

## Abstract

Type-A and -B GABA receptors (GABA_A_Rs/GABA_B_Rs) control brain function and behaviour by fine tuning neurotransmission. Over-time these receptors have become important therapeutic targets for treating neurodevelopmental and neuropsychiatric disorders. Several positive allosteric modulators (PAMs) of GABARs have reached the clinic and selective targeting of receptor subtypes is crucial. For GABA_B_Rs, CGP7930 is a widely used PAM for *in vivo* studies, but its full pharmacological profile has not yet been established. Here, we reveal that CGP7930 has multiple effects not only on GABA_B_Rs but also GABA_A_Rs, which for the latter involves potentiation of GABA currents, direct receptor activation, and also inhibition. Furthermore, at higher concentrations, CGP7930 also blocks G protein-coupled inwardly-rectifying K^+^ (GIRK) channels diminishing GABA_B_R signalling in HEK 293 cells. In male and female rat hippocampal neuron cultures, CGP7930 allosteric effects on GABA_A_Rs caused prolonged rise and decay times and reduced the frequency of inhibitory postsynaptic currents and potentiated GABA_A_R-mediated tonic inhibition. Additional comparison between predominant synaptic- and extrasynaptic-isoforms of GABA_A_R indicated no evident subtype selectivity for CGP7930.

In conclusion, our study of CGP7930 modulation of GABA_A_Rs, GABA_B_Rs and GIRK channels, indicates this compound is unsuitable for use as a specific GABA_B_R PAM.

## Highlights

• CGP7930, an established GABA_B_R PAM, inhibits GABA_B_R signalling at >3 μM concentration.
• CGP7930 apparent inhibitory activity at GABA_B_Rs results from inhibition of GIRK channels.
• CGP7930 is also an efficacious and potent GABA_A_R ligand.
• CGP7930 is approximately equipotent at GABA_A_Rs and GABA_B_Rs.
• Physiological actions of CGP7930 will involve an array of targets including type-A and -B GABARs and K^+^ channels.

## 1 Introduction

GABA-mediated inhibition shapes cellular and neural network signalling pathways that underlie brain function, including consciousness, executive decision making, cognition, and overall control of excitability, as well as mood and sleep. Unsurprisingly, dysfunction of GABAergic neurotransmission results in far-ranging neurological and psychiatric consequences (Möhler, 2006). At a cellular level, neuronal inhibition, which is the main activity for the neurotransmitter GABA in the central nervous system, is achieved by activating two distinct classes of GABA receptor: type-A and type-B. GABA_A_Rs are anion-permeable members of the pentameric ligand-gated ion channel family (Smart and Paoletti, 2012) whereas GABA_B_Rs are class C G-protein coupled receptors (GPCRs) that signal variously via Gα_i/o_ to: activate G protein-coupled inwardly-rectifying K^+^ channels (GIRKs); inhibit Ca^2+^ channels; and inhibit adenylyl cyclase activity (Bettler and Tiao, 2006). The combined actions of these two receptor systems are pivotal for controlling neural network output and behaviour.

Given the impact of GABAergic inhibition on neurophysiology, ligands targeting GABA receptors are frequently employed to treat brain disorders (Bettler and Tiao, 2006; Sieghart and Savi, 2018). For many years, the GABA_B_R specific agonist baclofen has been used to treat spasticity, epilepsy, substance abuse, addiction and alcoholism (Froestl, 2010). However, due to off-target effects, low brain permeability and tolerance, several positive allosteric modulators (PAMs) of GABA_B_Rs have been developed and these have become pre-eminent in GABA_B_R-related drug discovery programmes. Among these, 3-(3′,5′-Di-tert-butyl-4′-hydroxy) phenyl-2,2-dimethylpropanol (CGP7930; Urwyler et al., 2001) is a highly characterised PAM and has been used extensively in animal models for treating anxiety and depression (Frankowska et al., 2007; Jacobson and Cryan, 2008), epilepsy (Mareš, 2012), alcoholism (Maccioni and Colombo, 2009), substance abuse (Smith et al., 2004), psychosis and schizophrenia (Ma and Stan Leung, 2017; Wierońska et al., 2015), pain (Brusberg et al., 2009), sedation (Carai et al., 2004) and nicotine dependence (Paterson et al., 2008), which together, support a role(s) for GABA_B_Rs in these disorders. However, its structural similarity to the general anaesthetic propofol (Parker et al., 2011, Fig. 1A) and ability to stimulate biochemical pathways in cells lacking GABA_B_Rs (Olianas et al., 2017), strongly suggests it has additional targets. Overall, even though the *in vivo* activity of CGP7930 has been widely studied, the full pharmacological profile of this ligand has not been established.

**Fig. 1:**
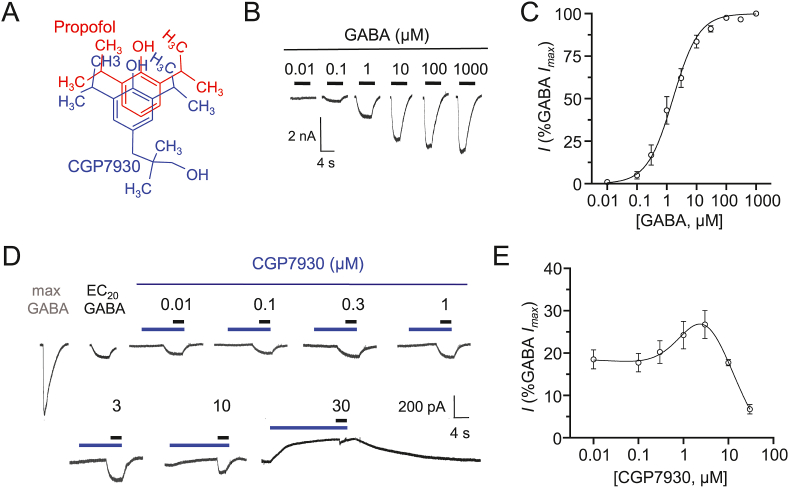
Modulatory effects of CGP7930 on GABA_A_ and GABA_B_ receptors *A)* Comparative overlay for CGP7930 (blue) and propofol (red) structures reveals a high degree of conformational similarity. *B)* Whole cell GABA current profiles recorded from GIRK cells expressing GABA_B_ receptors activated by 0.01–1000 μM GABA. *C)* Normalised GABA concentration-response relationship for GABA_B_ receptor activated currents in GIRK cells. (EC_50_ = 2.0 ± 0.7 μM; n = 6). *D)* Representative whole-cell currents activated by ∼ EC_20_ GABA (black bar) in the absence and presence (following pre-incubation) of CGP7930 (blue bar). *E)* Concentration-response relationship for CGP7930 modulating EC_20_ GABA_B_ receptor activated currents normalised (%) to the control maximal GABA response in the absence of CGP7930 (EC_50_ = 11.1 ± 7.0 μM; IC_50_ = 10.5 ± 2.0 μM; n = 4). Note the bell-shaped nature of the curve. In this and succeeding figures, concentration response data points represent the mean ± SEM. (For interpretation of the references to colour in this figure legend, the reader is referred to the web version of this article.)

Given CGP7930's structural resemblance to propofol, a GABA_A_R PAM, these receptors were first considered in this study as a potential additional target. Although GABA_A_Rs are assembled from 19 subunits (α1-6, β1-3, γ1-3, ρ1-3, δ, ε, θ, π), only a relatively restricted number of subunit combinations are thought to be expressed in the brain (Sieghart and Sperk, 2002). Thus, prototypical synaptic GABA_A_Rs are comprised of 2α, 2β and a γ subunit (Laverty et al., 2019), whilst the extrasynaptic αβδ receptors may exhibit greater stoichiometric variation (Kasaragod et al., 2022; Sente et al., 2022; Sieghart and Sperk, 2002). These receptors mediate neuronal inhibition at inhibitory synapses generating transient (ms) increases in membrane conductance, and by initiating tonic inhibition (due to low prolonged ambient GABA levels), causing persistent electrical shunting of excitatory synaptic potentials (Farrant and Nusser, 2005). In concert, these two types of inhibition exert a profound influence over excitatory neurotransmission (Mitchell and Silver, 2003). Depending on the brain region, phasic and tonic inhibition can be mediated by distinct subpopulations of GABA_A_Rs. For example, while α1/2β2/3γ2 receptors mediate the vast majority of synaptic inhibition in the neocortex, α5β3γ2 and α4β3δ receptors mediate most of the tonic inhibition in this region (Hutcheon et al., 2004).

Due to the paucity of studies characterising the pharmacological properties of CGP7930 in native neuronal tissue and heterologous expression systems, we utilised a range of strategies to demonstrate that CGP7930 modulates both GABA_A_Rs and GABA_B_Rs, as well as GIRK channels. Together, this provides new insight into the perceived mechanism of action for CGP7930.

## 2 Materials and methods

### 2.1 cDNAs, plasmids and drugs

cDNAs for eGFP and murine GABA_A_R α1, α4, β2/3, γ2L and δ subunits and GABA_B_R R1a and R2 subunits, cloned into a pRK5 vector, have been described previously (Hannan et al., 2011, 2019). CGP7930 and (RS) - baclofen were acquired from Tocris. CGP55845, and other drugs, were obtained from Merck (Sigma-Aldrich) unless otherwise stated.

### 2.2 Cell culture and transfection

HEK-293T cells were maintained in Dulbecco's modified Eagle's medium containing 10% (v/v) fetal calf serum (FCS), 100 U/ml penicillin-G, 100 μg/ml streptomycin in 95% air/5% CO_2_ at 37 °C. All cell culture reagents were acquired from ThermoFisher unless otherwise stated. Cells were plated on 22 mm glass coverslips (VWR, UK), coated with poly-l-lysine (Sigma) and transfected using a calcium phosphate method with cDNAs for eGFP, α, β, γ or δ, in a 1:1:1:1 ratio (Hannan et al., 2020) applied 1–2 h after cell plating. HEK-293 cells stably expressing Kir3.1/3.2 channels (Leaney et al., 2000) were grown in a selection media containing G418 (0.5 mg/ml) and Zeocin (0.4 mg/ml) and passaged, and plated, as for the HEK-293T cells above. These cells are referred to as GIRK cells throughout the text and were transiently transfected with cDNAs for eGFP, GABA_B_ R1a and R2 in a 1:1:5 ratio applied 1–2 h after cell plating.

### 2.3 Neuronal cultures

All animal studies were carried out in accordance with the UK Animals (Scientific Procedures) Act, 1986. Dissociated hippocampal cultures were prepared from male and female embryonic day 18 Sprague-Dawley rats as described previously (Hannan et al., 2020). Briefly, single cells derived from dissected hippocampi were seeded onto glass coverslips coated with poly-d-lysine in a plating medium containing minimum essential media with 5% v/v heat-inactivated FCS, 5% v/v heat-inactivated horse serum, penicillin-G/streptomycin (100 U/100 μg/ml), 2 mM l-glutamine, and 20 mM glucose. Two hr after plating the media was changed to a Neurobasal-A based maintenance media supplemented with 1% v/v B-27, penicillin-G/streptomycin (100 U/100 μg/ml), 0.5% v/v Glutamax and 35 mM glucose. Neurons were grown at 37 °C in humidified 95% air/5% CO_2_.

### 2.4 Whole-cell patch-clamp electrophysiology

GABA-activated currents were recorded 24 h after transfection of HEK-293T or GIRK cells. Spontaneous inhibitory postsynaptic currents (sIPSCs) were recorded from cultured hippocampal neurons at 12–17 days *in vitro* (*DIV)*. Cells were superfused with a saline solution containing (mM): 140 NaCl, 4.7 KCl, 2.52 CaCl_2_, 1.2 MgCl_2_, 11 glucose, and 5 HEPES; pH 7.4. sIPSCs from hippocampal culture neurons were recorded in the presence of 2 mM kynurenic acid to block excitatory neurotransmission. For recording K^+^ currents in GIRK cells and neurons, the bath solution was switched to a high K^+^ solution containing 25 mM KCl and reduced NaCl (120 mM), to shift the equilibrium potential for K^+^ from approximately −90 to −47 mV, thus reversing the direction of K^+^ current flux to net inward. GABA_B_R-activated K^+^ currents in cultured neurons were recorded in the additional presence of 2 mM kynurenic acid and 20 μM picrotoxin.

Borosilicate glass electrodes (3–5 MΩ) were filled with a K^+^-based internal solution for recording GIRK currents containing (mM): 120 KCl, 2 MgCl_2_, 11 EGTA, 30 KOH, 10 HEPES, 1 CaCl_2_, 1 GTP, 2 ATP, 14 creatine phosphate, pH 7.0. GABA_A_R currents and sIPSCs were recorded with a Cs-based internal solution containing (mM): 120 CsCl, 1 MgCl_2_, 11 EGTA, 30 KOH, 10 HEPES, 1 CaCl_2_, and 2 K_2_ATP; pH 7.2. All internal solutions were adjusted to approximately 305 mOsm/l. Cells were held at −60 mV (sIPSCs) or −60/−70 mV (K^+^ currents). Voltage clamp recordings were undertaken after optimising series resistance (Rs, <10 MΩ) and compensating for the whole-cell membrane capacitance. Membrane currents were filtered at 5 kHz (−3 dB, 6th pole Bessel, 36 dB/octave) and stored for analysis with Clampex 10.

Concentration-response curves were generated by measuring the current (*I*) for each ligand concentration and normalising to the maximal current response (*I*_max_). Data fitting was performed with a Hill equation:

*I* = *I*_min_ + (*I*_*max*_*-I*_*min*_)(1 / (1 + (EC_50_ / [A])^n^)

where *I*_min_ defines the pedestal current response, A is the concentration of the agonist, EC_50_ is the concentration of agonist giving 50% of the maximum response and n is the Hill slope.

For the biphasic curve fits, the following modified Hill equation was used:

*I* = *I*_min_ + (*I*_*max*_*-I*_*min*_)*((1 / (1 + (EC_50_ / [A])^n^))-(1 / (1 + (IC_50_/[A])^m^)))

Where IC_50_ defines the concentration of ligand causing a 50% reduction in the maximal current with a Hill slope of m (Halliwell et al., 1999).

sIPSCs were recorded at room temperature (20–23 °C) and detected using WinEDR and WinWCP (Strathclyde Electrophysiology Software UK), and frequency was calculated for 60 s recording epochs. For sIPSC amplitudes, several hundred events were recorded per condition and analysed as an average amplitude per cell.

For kinetic analysis, individual uncontaminated sIPSCs were isolated and the average 10–90% rise time and exponential decay times were measured from the mean sIPSC waveform. Weighted decay times are reported encompassing mono- and bi-exponentially decaying events according to the equation:

τ_w_ = (A_1_ *τ_1_ + A_2_ *τ_2_) / (A_1_ + A_2_)

where τ_1_ and τ_2_ are exponential decay time constants, and A_1_ and A_2_ are the relative amplitude contributions of τ_1_ and τ_2_, respectively.

For tonic inhibition, to determine the average holding currents, a 30 s continuous holding current recording was sampled every 1 s, discarding epochs that coincided with sIPSCs. Any effect of CGP7930 or bicuculline on the holding current was defined by subtracting the average holding currents in control and during drug application. The baseline root-mean-square current variance (RMS) was calculated before and during drug treatment. This was estimated from a continuous (20 s) current recording, sampled every 100 ms. The median current was calculated every 5 s and values more than twice the standard deviation from the median (usually due to IPSCs) were eliminated. All drugs were applied using a U-tube rapid drug application system, or more slowly via bath perfusion.

### 2.5 Statistics

All statistical tests were performed in GraphPad Prism and sample sizes are indicated in the figure legends and results. Data were subjected to a test for normality using the Kolmogorov-Smirnov test. Outliers were identified using ROUT (Q = 1%). We did not make any systematic allowance for heteroscedasticity. Curve fits were spot-checked by weighting each point according to the (SD)^−1^ and this did not affect our conclusions or the curve fits.

For normally-distributed dataset comparisons, we used a one-way ANOVA with Dunnett's multiple comparisons test. The bar chart data represent mean ± SEM. The data contained in the box plots show the median, 5–95% whiskers and 25–75% interquartile ranges.

## 3 Results

### 3.1 CGP7930 is a potent positive allosteric modulator of GABA_B_ receptors

The pharmacological profile of CGP7930 at GABA_B_Rs was initially characterised by analysing GABA-activated K^+^ currents in HEK cells stably expressing the inward rectifier K^+^ channels, Kir3.1 and 3.2, and transiently transfected with GABA_B_ R1a and R2 subunits (termed GIRK cells). These cells were bathed in high external K^+^ concentration Krebs to reverse the direction of net K^+^ current flow. GABA_B_Rs were activated with the natural transmitter, GABA (EC_50_ = 2.0 ± 0.7 μM; n = 6; Fig. 1B–C). The extent of CGP7930 modulation at GABA_B_Rs was revealed by pre-applying CGP7930 to GIRK cells until the response reached a plateau followed by co-application with ∼EC_20_ GABA (Fig. 1D–E). The threshold for GABA current potentiation was apparent between 0.1 and 0.3 μM CGP7930, reaching nearly 30% of the maximum GABA current activation of GABA_B_Rs, with no indication of direct receptor activation (Fig. 1D). However, at 1 μM, and very clearly at 10–30 μM, CGP7930 caused a slow outward current that substantially diminished the GABA response and caused the CGP7930 PAM concentration response curve to become bell-shaped, indicative of inhibition (Fig. 1D–E).

**Fig. 2:**
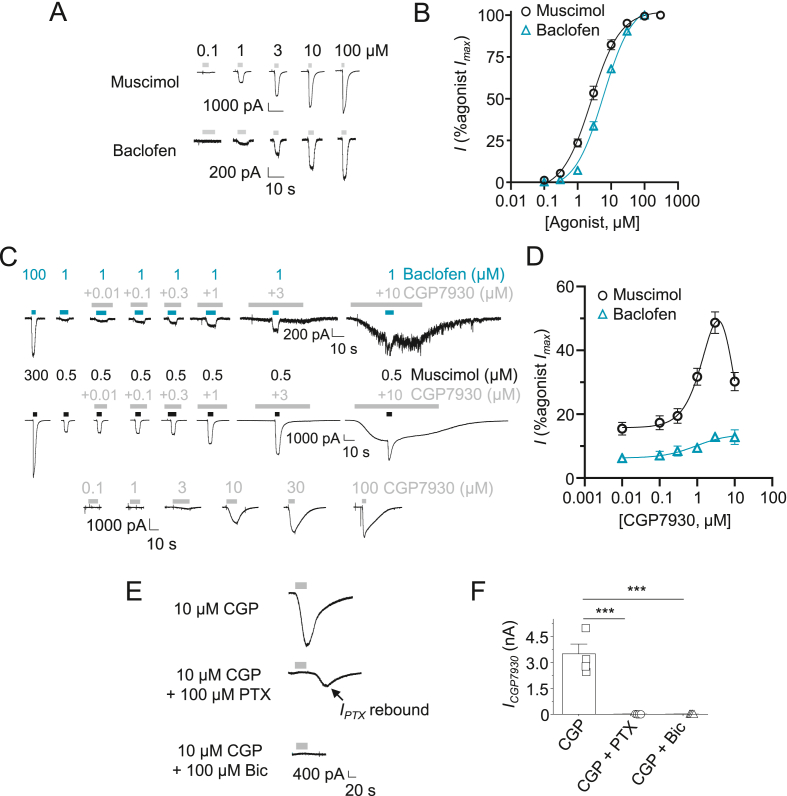
CGP7930 modulation of neuronal GABA_A_ and GABA_B_ receptors *A)* Whole-cell voltage-clamp baclofen- and muscimol-activated currents recorded from hippocampal neurons in culture. *B)* Normalised concentration-response relationships for baclofen and muscimol in hippocampal neurons. Baclofen EC_50_ = 5.6 ± 0.4 μM (n = 12) and muscimol EC_50_ = 2.7 ± 0.1 μM (n = 9). *C)* Whole-cell currents in hippocampal cultured neurons evoked by maximum and ∼EC_10-15_ baclofen (1 μM, top panel, blue bar) and muscimol (0.5 μM, middle, black bar) showing potentiation by CGP7930 (pre-incubation, grey bar), and direct activation by CGP7930 when applied alone (bottom). Note that cells were bathed in high external K^+^ for recording baclofen-activated currents and for the direct activation by CGP7930. *D)* Baclofen and muscimol agonist potentiation-response curves in the presence of CGP7930, normalised (%) to the respective control maximum agonist response (*I*_max_) (Muscimol: EC_50_ = 2.0 ± 1.1 μM; IC_50_ = 7.9 ± 1.8 μM; n = 10; Baclofen: EC_50_ = 0.9 ± 0.6 μM; n = 7). *E)* Whole-cell voltage-clamp currents directly activated by 10 μM CGP7930 in high K^+^ external solution. The inward current is blocked by either 100 μM picrotoxin (PTX) or bicuculline (Bic). Note that the downward deflection after picrotoxin application is a rebound current arising post wash-off. *F)* Bar chart showing inhibition of CGP7930 direct activation current by PTX and Bic (both 100 μM). Bar graph represents the mean ± S.E.M. n = 4–12 cells; ***P < 0.001; one-way ANOVA. (For interpretation of the references to colour in this figure legend, the reader is referred to the web version of this article.)

CGP7930 modulation at neuronal GABA_B_Rs was also characterised using baclofen-activated GIRK currents in hippocampal cultures similarly bathed in a high external K^+^ concentration. Receptors were activated with ∼EC_10-15_ baclofen (EC_50_ = 5.6 ± 0.4 μM; n = 12; Fig. 2A–B) in the presence of 2 mM kynurenic acid to block excitatory postsynaptic currents (EPSCs) and 20 μM picrotoxin to block GABA_A_R-mediated inhibitory postsynaptic currents (IPSCs). Constructing PAM concentration response-curves by pre-applying CGP7930 to neurons for 10 s, followed by co-application with ∼EC_10-15_ baclofen (1 μM), revealed the extent of CGP7930 modulation of GABA_B_Rs (Fig. 2C–D). By contrast with GIRK cells, at concentrations >3 μM, pre-application of CGP7930 caused a slow inward current (Fig. 2C). For >10 μM CGP7930, this slow inward current masked the baclofen-activated currents. Furthermore, no outward current was seen, as observed previously in GIRK cells. As a result, the CGP7930 PAM concentration-response curve was shallow and showed only a small potentiation of the baclofen response. The additional slow inward current observed in neurons, (but not in GABA_B_R expressing GIRK cells), caused by > 3 μM CGP7930 (Fig. 2C), was abolished by 100 μM picrotoxin (P < 0.001) and by another GABA_A_R selective antagonist bicuculline (100 μM; P < 0.001: Fig. 2E–F. This would suggest that CGP7930 is also modulating (and/or possibly directly activating) GABA_A_Rs over a similar concentration range to that for GABA_B_Rs.

To further investigate CGP7930 modulation of GABA_A_Rs in hippocampal neurons, we used the specific type-A receptor agonist muscimol (Fig. 2A–D) with a Cs^+^-based internal solution to block GIRK channels (Gahwiler and Brown, 1985) under physiological external K^+^ levels. As previously observed, CGP7930 alone evoked a slowly deactivating current in hippocampal neurons (Fig. 2C) with an EC_50_ of 5.2 ± 0.1 μM, achieving a maximal direct activation of 42.1 ± 1.8% (n = 8; concentration response curve not shown) compared to the maximal GABA_A_R activation caused by a saturating concentration of muscimol. Pre-application of CGP7930 for 10 s followed by co-application with ∼EC_10-15_ muscimol (in the presence of kynurenic acid) revealed a concentration-dependent potentiation of GABA_A_R currents, with the potentiation curve declining at the highest concentration of CGP7930 tested (10 μM). This resulted in a bell-shaped curve after the direct effect of CGP7930 was subtracted (Fig. 2C–D; CGP7930 EC_50_ = 2.0 ± 1.1 μM; IC_50_ = 7.9 ± 1.8 μM; n = 10). Overall, these results indicate a strong modulation of native GABA_A_Rs by CGP7930.

### 3.2 CGP7930 is an inhibitor of inwardly-rectifying potassium channels

**Fig. 3:**
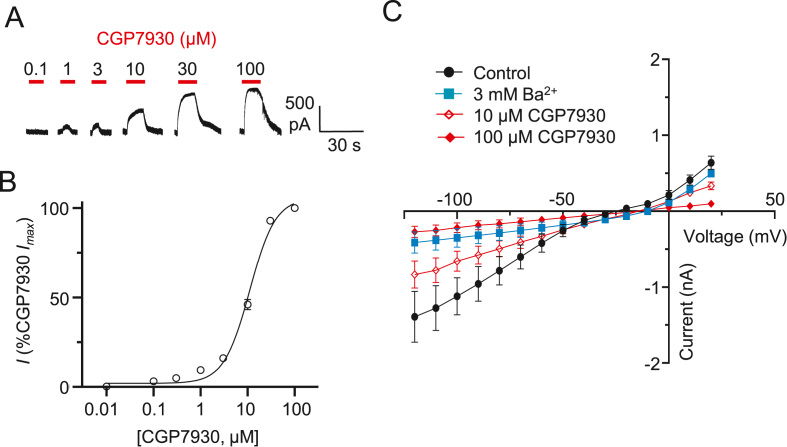
Inhibition of K^+^ currents by CGP7930 in GIRK cells *A)* Representative, basally-active, whole-cell currents for GIRK channels bathed in high K^+^ solution, showing inhibition by CGP7930 (0.1–100 μM). Note, the inward basal K^+^ current is revealed following CGP7930 block. *B)* Concentration response curve for CGP7930 blocking basally-active GIRK channels shown as increasing outward K^+^ currents that are normalised to the maximal (%) CGP7930 block (= maximum outward current response) from the same cell. EC_50_ = 9.7 ± 0.6 μM; n = 4. *C)* Current-voltage relationships (I–V) for basally active whole-cell K^+^ currents in GIRK cells under the following conditions: control/basal I–V (black); +3 mM Ba^2+^ (blue); +10 μM CGP7930 (red open symbols); and +100 μM CGP7930 (red filled symbols); n = 5–6. (For interpretation of the references to colour in this figure legend, the reader is referred to the web version of this article.)

We hypothesised that the previously observed slow outward current caused by CGP7930 in GIRK cells (Fig. 1D) might be due to an inhibition of the inwardly-rectifying K^+^ channels expressed in these cells. To examine this possibility, we applied CGP7930 (0.01–100 μM) to GIRK cells, not expressing GABA_B_Rs, voltage clamped at −60 mV whilst bathed in high K^+^ external Krebs. Whole-cell outward currents were evident at a threshold of 0.3 μM CGP7930, approaching a peak at 100 μM and were readily reversible on washout of CGP7930 (Fig. 3A). The potency of CGP7930 for generating the outward current was lower than that for CGP7930 potentiation at GABA_B_Rs, with an EC_50_ of 9.7 ± 0.6 μM (Fig. 3B). Our recording conditions, using high external K^+^ Krebs, suggested the outward currents caused by CGP7930 are likely to be due to a block of basally-activated K^+^ channels in the GIRK cells. The voltage-dependent nature of these currents was explored by constructing a current-voltage (I–V) relationship (Fig. 3C). Applying 10 mV step changes in the holding potential revealed GIRK-mediated inwardly-rectifying currents between −20 and −120 mV. Application of 10 or 100 μM CGP7930 caused inhibition of these currents, a feature also seen with the inward-rectifier blocker, Ba^2+^ (Hagiwara et al., 1978; Standen and Stanfield, 1978) applied at 3 mM (Fig. 3C). As suggested above, this is likely to involve inhibition of basally active Kir3.1/3.2 that are expressed by the GIRK cells. Application of depolarising 10 mV steps revealed evidence of additional outward rectification. This was also inhibited by CGP7930, but not by Ba^2+^, uncovering another voltage-sensitive channel population as a target for CGP7930 (Fig. 3C).

### 3.3 CGP7930 is a potent positive allosteric modulator of GABA_A_ receptors

**Fig. 4:**
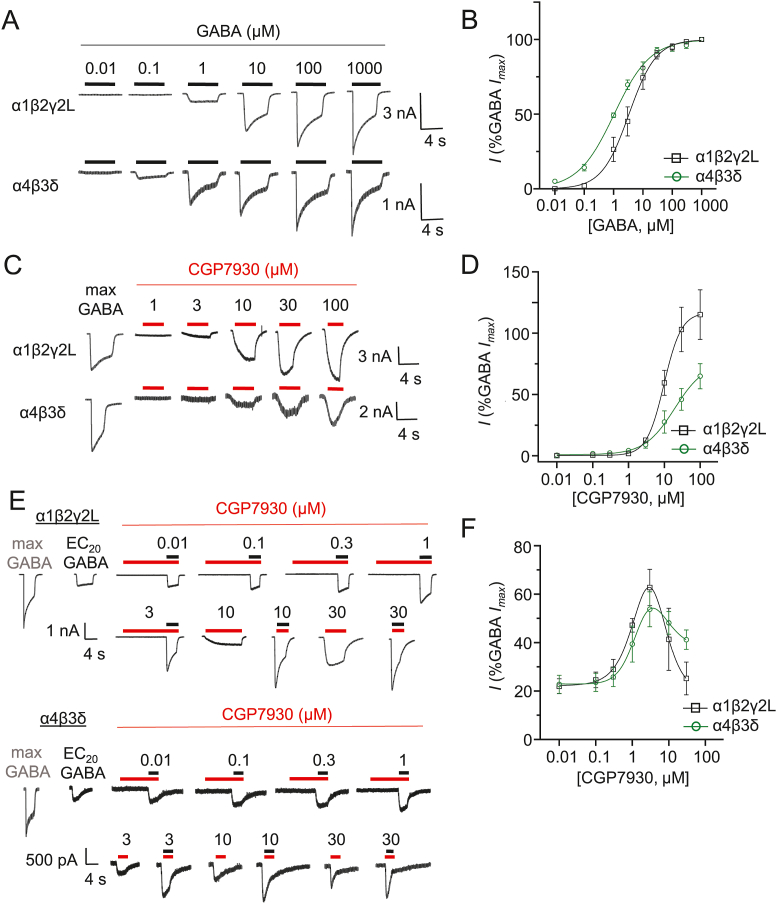
Modulatory effects of CGP7930 at recombinant GABA_A_ receptor currents in HEK-293 cells *A)* Whole-cell GABA-activated current profiles recorded from HEK-293 cells expressing α1β2γ2L (top) or α4β3δ (bottom) receptors. *B)* GABA concentration-response relationships for α1β2γ2L and α4β3δ receptors expressed in HEK-293 cells. EC_50_ α1β2γ2L = 5.1 ± 1.7 μM; n = 9; α4β3δ = 1.2 ± 0.1 μM; n = 7. *C)* Directly-activated CGP7930 currents (1–100 μM, red bars) recorded from HEK-293 cells expressing either α1β2γ2L or α4β3δ receptors. *D)* Concentration-response curves for CGP7930 direct activation of α1β2γ2L or α4β3δ receptors (normalised to the respective maximum GABA currents). *E)* Control maximum and ∼EC_20_ GABA (black) currents recorded from HEK-293 cells expressing α1β2γ2L and α4β3δ receptors, followed by GABA EC_20_ currents in the pre-applied presence (red bar) of 0.01–30 μM CGP7930. Note direct activation currents for 3, 10 and 30 μM CGP7930 are also shown, prior to co-application with GABA EC_20_. *F)* GABA EC_20_ modulation curves for α1β2γ2L and α4β3δ in the presence of CGP7930 normalised to the respective maximum GABA currents; (α1β2γ2L: EC_50_ = 1.7 ± 1.3 μM; IC_50_ = 6.6 ± 2.3 μM; n = 5; α4β3δ: EC_50_ = 1.0 ± 0.3 μM; IC_50_ = 19.6 ± 13.1 μM n = 7). (For interpretation of the references to colour in this figure legend, the reader is referred to the web version of this article.)

The slow inward current caused by CGP7930 in cultured neurons and its block by both picrotoxin and bicuculline clearly implicated the involvement of GABA_A_Rs. We explored the ability of CGP7930 to modulate different recombinant GABA_A_Rs expressed in HEK-293 cells to avoid confounds arising from heterogeneous receptor subtypes in neurons. Two receptor isoforms were selected for study - the α1β2γ2L receptor, considered to be a major synaptic GABA_A_R isoform throughout the central nervous system, and α4β3δ which is a recognised extrasynaptic GABA_A_R in the thalamus and hippocampus (Olsen and Sieghart, 2009; Sieghart and Sperk, 2002). GABA concentration-response curves in the absence of CGP7930 revealed EC_50_s of 5.1 ± 1.7 μM and 1.2 ± 0.1 μM, respectively, in accord with those previously reported (Mortensen et al., 2011, Fig. 4A–B). Application of CGP7930 alone was able to directly activate either GABA_A_R isoform with a threshold of ∼1–3 μM (Fig. 4C–D). The macroscopic efficacy at α1β2γ2L receptors was greater than for α4β3δ receptors (Fig. 4D), although the EC_50_s for CGP7930 were similar (9.3 ± 0.7 μM (αβγ), 13.1 ± 1.4 μM (αβδ)).

Pre-application of CGP7930 to α1β2γ2L receptors revealed potentiation of the GABA current at a threshold of 1 μM CGP7930 just before clear direct activation of the receptor by CGP7930 became evident at approximately 3–10 μM (Fig. 4E). Subtracting the direct effects observed for 3–30 μM CGP7930 from the co-applied ∼ EC_20_ GABA/CGP7930-activated current revealed a bell-shaped CGP7930 concentration-response curve (Fig. 4F). The profile of the CGP7930 potentiation curve for α1β2γ2L receptors was also reflected in similar experiments conducted with α4β3δ receptors, suggesting there was no GABA_A_R subtype selectivity for CGP7930 between these isoforms. The descending component of the CGP7930 potentiation curve has several potential interpretations. It may indicate some signalling commonality between receptor activation and potentiation, such that potentiation is limited by high levels of receptor activation; or it could indicate a degree of receptor block/inhibition by CGP7930, or an increased level of GABA_A_R desensitisation. These results show that CGP7930 is a significant allosteric modulator at GABA_A_Rs at a concentration range that overlaps with the modulation of GABA_B_Rs.

### 3.4 CGP7930 reduces sIPSC frequency in hippocampal neurons

Having established the allosteric modulation of GABA_A_Rs by CGP7930, and even though we also established CGP7930 has multiple targets, we assessed its direct functional effects on GABA_A_R-mediated inhibition using hippocampal whole-cell recording in the presence of the GABA_B_R antagonist CGP55845 (1 μM) which, under our recording conditions, has an IC_50_ of 0.01 ± 0.0007 μM (n = 6; data not shown). Initially, we examined phasic inhibition by monitoring the frequency and amplitude of sIPSCs, which in the hippocampus, will mostly arise from synaptic receptors comprising α1/2β2/3γ2 subunits (Datta et al., 2015; Hutcheon et al., 2004).

**Fig. 5:**
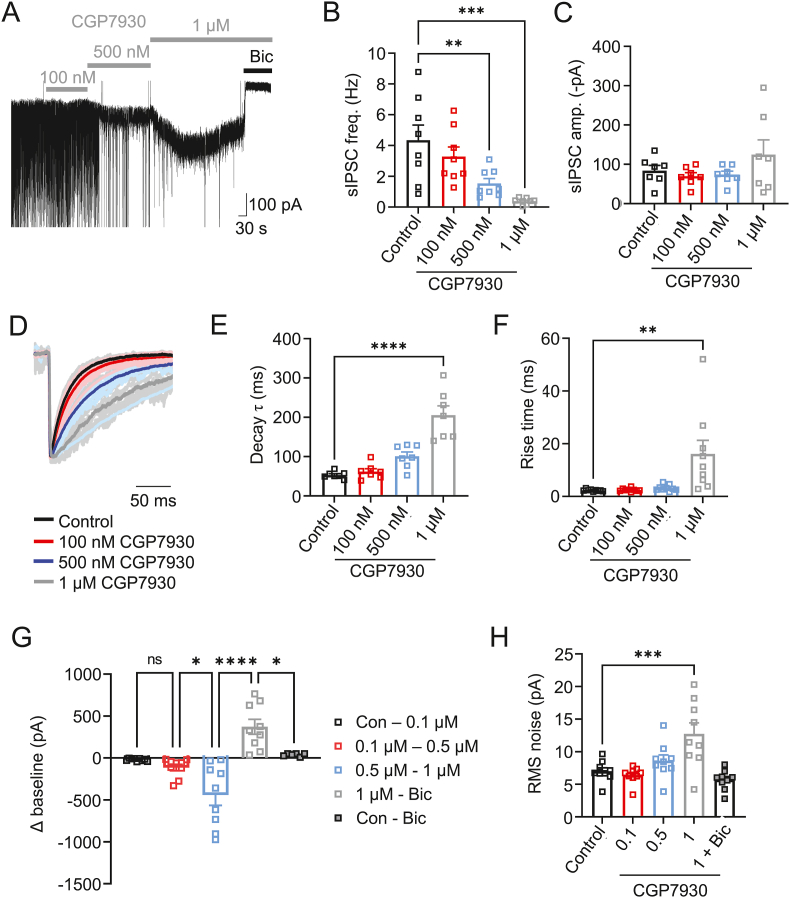
Modulatory effects of CGP7930 on phasic and tonic inhibition in cultured hippocampal neurons *A)* Representative sIPSCs recorded from a hippocampal cultured neuron in control conditions and in the presence (bars) of CGP7930 (0.1–1 μM). Note sIPSC amplitudes are ‘clipped’ in some panels to show details of CGP7930 direct effects. Bicuculline (Bic, 50 μM) was applied at the end of each experiment to block all IPSCs. *B, C)* Frequency (B) and amplitude (C) bar graphs of sIPSCs in hippocampal neurons in the absence (control) or presence (0.1–1 μM) of CGP7930. Individual data points are shown including the mean ± SEM. *D)* Individual and mean (bold) peak-scaled sIPSC waveforms recorded from hippocampal neurons are shown for control and in the presence of CGP7930 (0.1–1 μM). *E, F)* Bar graphs for IPSC exponential decay times (E) and 10–90% rise times (F) for hippocampal neurons. *G, H)* CGP7930-induced changes to the baseline holding currents (G) and mean RMS current noise (H) for hippocampal neurons. Relative changes to the baseline holding current and RMS noise are shown on moving sequentially from one condition, ie, control, to another condition, ie, 0.1 μM CGP7930. Individual data points and mean ± SEM are shown. n = 7–9 cells, ns – not significant; *P < 0.05, **P < 0.01, ***P < 0.001, ****P < 0.0001; One-way ANOVA with Dunnett's multiple comparison test.

Applying 0.1, 0.5 and 1 μM CGP7930 reduced sIPSC frequency in a concentration-dependent fashion (p = 0.0075 at 0.5 μM, p = 0.0002 at 1 μM, F_(3, 28)_ = 8.5, p = 0.0003, One-way ANOVA) while the mean sIPSC amplitude remained unchanged (F_(3, 24)_ = 1.431, p = 0.258, One-way ANOVA; Fig. 5A–C). These results initially suggested that CGP7930 does not alter the postsynaptic organisation of GABA_A_Rs and that the profound change to sIPSC frequency most likely reflects reduced GABA release from interneurons, possibly due to CGP7930 modulating the activity of presynaptic GABA_A_Rs. Slow inward currents were also evident with 0.5 and 1 μM CGP7930, which were abolished by bicuculline (Fig. 5A).

### 3.5 CGP7930 reduces the decay rate for GABAergic sIPSCs

The binding of PAMs, such as neurosteroids and benzodiazepines, to the GABA_A_R, influences neuronal activity by altering the kinetics of synaptic GABA_A_Rs, leading to prolonged IPSC decay times and increased inhibitory charge transfer (Belelli and Lambert, 2005; Perrais and Ropert, 1999). Characterising the effect of CGP7930 on the kinetics of sIPSCs in hippocampal neurons also revealed prolonged sIPSC decay times in a concentration-dependent manner compared to control (Decay τ: p = 0.054 at 500 nM, P < 0.0001 at 1 μM, F_(3,24)_ = 26.33, P < 0.0001, One-way ANOVA; Fig. 5D–E). The rise time of sIPSCs was also slowed in CGP7930 compared to controls (p = 0.002 at 1 μM, F_(3,32)_ = 6.729, p = 0.0012, One-way ANOVA; Fig. 5F; Table 1).

**Table 1:** Phasic inhibition - kinetic properties of sIPSCs in CGP7930.

			Frequency (Hz)	Amplitude (pA)	Decay τ (ms)	Rise time (ms)
Hippocampal neurons (cultures)	Control		4.35 ± 0.98 (8)	−83.9 ± 13.2 (7)	53 ± 3.3 (7)	2.3 ± 0.14 (9)
	CGP7930	100 nM	3.28 ± 0.62 (8)	−70.0 ± 8.9 (7)	61.9 ± 7.2 (7)	2.5 ± 0.20 (9)
		500 nM	1.53 ± 0.32 (8)	−73.9 ± 8.8 (7)	100.8 ± 11 (7)	3.2 ± 0.39 (9)
		1 μM	0.38 ± 0.07 (8)	−124.6 ± 37.7 (7)	205.5 ± 117 (7)	16.1 ± 5.2 (9)

Table 1 - Phasic inhibition: kinetic properties of sIPSCs in control and after CGP7930, reporting mean sIPSC frequency, amplitude, weighted decay time and the rise-time. Kinetic properties of sIPSCs are shown as mean ± SEM. Number of cells are indicated in parentheses.

These results suggest that CGP7930 can potentiate GABAergic synaptic inhibition by prolonging the activation and decay kinetics of synaptic GABA_A_Rs, but most likely the main effect is the reduced sIPSC frequency. It is noteworthy that multiple kinetic parameters for sIPSCs are modulated by micromolar concentrations of CGP7930.

### 3.6 CGP7930 increases tonic inhibition in hippocampal neurons

Tonic inhibition is another important component of GABAergic inhibition mediated mostly by extrasynaptic receptors. In the hippocampus, two distinct receptor subtypes are mainly involved. In dentate gyrus granule cells, tonic inhibition derives largely from α4β3δ receptors, whereas for hippocampal CA1 pyramidal neurons, α5β2/3γ2 receptors predominate, supported by a smaller population of αβ di-heteromeric receptors (Farrant and Nusser, 2005; Mortensen and Smart, 2006).

We assessed the effect of CGP7930 on tonic inhibition in hippocampal cultured neurons. Application of CGP7930 caused a concentration-dependent increase in baseline holding currents (P < 0.001 at 1 μM, F_(4,37)_ = 15.85, P < 0.0001, One-way ANOVA; Fig. 5G; Table 2), consistent with changes to the holding current during CGP7930 pre-application while constructing PAM concentration-response curves (Fig. 2C and D). Applying bicuculline reversed the 1 μM CGP7930 increase in tonic current back to control levels (Fig. 5G; P < 0.05; One-way ANOVA). The CGP7930-mediated increase in tonic current occurred concurrently with increments in the root-mean-square (RMS) for membrane current variance. This was also concentration-dependent and reversed by 50 μM bicuculline (P < 0.001 at 1 μM, F_(4,40)_ = 9.04, P < 0.0001, One-way ANOVA; Fig. 5H; Table 2).

**Table 2:** Tonic currents and RMS noise in CGP7930.

	Control to 100 nM	100–500 nM	500 nM to 1 μM	1 μM to Bic	Control to Bic
**Tonic currents (pA)**	−21.41 ± 5.4 (9)	−115.6 ± 37.8 (9)	−441.6 ± 124.7 (9)	372.0 ± 88.5 (9)	38.02 ± 7.43 (6)
	**Control**	**100 nM**	**500 nM**	**1 μM**	**1 μM + Bic**
**Membrane current RMS noise (pA)**	7.0 ± 0.5 (9)	6.4 ± 0.4 (9)	8.6 ± 0.9 (9)	12.7 ± 1.7 (9)	5.7 ± 0.5 (9)

Table 2 - Tonic currents and RMS noise in control and after application of CGP7930 and 50 μM bicuculline (Bic). Tonic current and RMS noise (current variance) values are shown as mean ± SEM. Numbers of cells are indicated in parentheses.

## 4 Discussion

CGP7930 has been widely used as an allosteric pharmacological tool to modulate GABA_B_Rs and circumvent the deleterious side-effects associated with the agonist baclofen. Despite characterising the biochemical and *in vivo* physiological properties of this PAM, there is a paucity of functional studies examining allosteric modulation of GABA_B_Rs by CGP7930 at a cellular level. While attempting to bridge this gap, we unexpectedly observed that a component of CGP7930 signalling is likely to be orchestrated via allosteric modulation of GABA_A_Rs and also inwardly rectifying K^+^ channels from the *KCNJ* sub-family (Alexander et al., 2021).

Although CGP7930 is capable of modulating both ionotropic and metabotropic GABA receptors over a similar dosage range, any potential value in this dual effect is compromised by other protein targets for CGP7930, not least the Kir3.1/3.2 channels that form part of the GABA_B_R signalling pathway. This very broad spectrum of activity indicates that the neuropharmacological actions for CGP7930 are very unlikely to be mediated solely by GABA_B_Rs. Initial evidence for CGP7930 acting as a PAM at GABA_A_Rs came from a fluorescent imaging assay using recombinant receptors expressed in HEK-293 cells (Sakamoto et al., 2019), though a direct comparison of CGP7930 modulation at type-A and -B GABA receptors in neurons was not explored. In the current study, by constructing PAM concentration response curves, using specific agonists for GABA_A_Rs and GABA_B_Rs, and by using the natural transmitter GABA, we were able to delineate receptor-specific signalling directly. From our findings, there appears to be no clear concentration ‘window’ for CGP7930 to selectively modulate just GABA_B_Rs without also modulating, or directly-activating, GABA_A_Rs, or inhibiting Kir3.1/3.2 channels.

Previous GABA_B_R studies using: [^35^S] GTPγS binding assays with brain lysates (Hensler et al., 2012) and CHO cells (Urwyler et al., 2001); inositol phosphate accumulation in HEK cells (Binet et al., 2004); c-AMP and adenylyl cyclase activity assays in neurons (Onali et al., 2003); voltage clamp recording from *Xenopus* oocytes and Ca^2+^ imaging assays in HEK cells, were used to track the effects of CGP7930 on GABA_B_R modulation (Urwyler et al., 2001). All these studies suggest that CGP7930 PAM activity at GABA_B_Rs has a threshold in the micromolar range. Our results are consistent with such a concentration range, but the important finding here is that CGP7930 also modulates GABA_A_Rs at similarly low overlapping concentrations. In addition, measuring the relative macroscopic efficacy of CGP7930, revealed significantly higher levels of potentiation at GABA_A_Rs compared to GABA_B_Rs, the latter most probably affected by the inhibition of the Kir channels. Overall, CGP7930 appears similar to other well-established PAMs of GABA_A_Rs, such as the benzodiazepines, propofol, and neurosteroids, in regard to its effective concentration range and scale of effect in neurons (Belelli and Lambert, 2005; Wang, 2011).

We confirmed the modulation and direct activation of GABA_A_Rs by CGP7930 by using the selective inhibitors bicuculline and picrotoxin, and by CGP7930 modulating heterologous recombinant GABA_A_Rs expressed in HEK-293 cells. The threshold concentration for modulating synaptic events (sIPSCs) was also comparable to those measured for both synaptic- and extrasynaptic-type recombinant GABA_A_Rs in HEK cells. Direct activation of GABA_A_Rs was also evident at concentrations of CGP7930 that are overlapping with those for modulating GABA_B_Rs. Therefore, it seems plausible that GABA_A_Rs are likely to be potentiated and directly-activated by concentrations of CGP7930 that are also modulating GABA_B_Rs.

Considering the physiological conditions under which GABA receptors are modulated by CGP7930, an equivalent role for this modulator at GABA_A_R- and GABA_B_R-based signalling seems highly likely. GABA_A_Rs localised at inhibitory synapses give rise to IPSCs and extrasynaptically localised GABA_A_Rs will underpin tonic inhibition. By contrast, GABA_B_Rs localised at perisynaptic areas of excitatory and inhibitory synapses will electrically shunt postsynaptic membranes (Kulik et al., 2002) and may contribute to tonic inhibition. Typically, GABA_B_R activation requires strong stimulation by released GABA following spillover (Isaacson et al., 1993; Scanziani, 2000). Therefore, under conditions of basal inhibitory neurotransmission, CGP7930 may not have a substantive effect on GABA_B_Rs, whereas potentiating and directly activating GABA_A_Rs that mediate phasic and tonic inhibition may be more significant. However, of note, CGP7930 also reduced the sIPSC frequency in our study, probably by reducing GABA release which may be achieved by activating presynaptic GABA receptors. This is likely to reduce GABA spillover, limiting both postsynaptic GABA_A_R and GABA_B_R activation.

CGP7930 is similar in structure to the general anaesthetic propofol (Parker et al., 2011) which binds to β-α and β-γ interfaces of GABA_A_Rs (Olsen, 2018). It is therefore possible that CGP7930 may bind to the same interfacial sites as propofol, although binding to other sites on GABA_A_Rs cannot be discounted. Interestingly, propofol can also interact with GABA_B_Rs (Xuan et al., 2018) reinforcing the notion that such similar chemical structures (e.g, CGP7930) might be expected to show promiscuous binding to GABA receptors.

These findings with CGP7930 serve as a cautionary note. The physiological actions of CGP7930 *in vivo* are extensively documented in numerous animal models. Most studies will have understandably applied only CGP7930 in the absence of baclofen as part of an experimental paradigm to reveal the impact of CGP7930 on phenotypes such as: substance abuse, addiction, drug-induced psychoses, seizures, analgesia, food intake or alcohol self-administration, and anxiety and depression. The outcomes from such studies will have been interpreted on the basis that CGP7930 selectively modulates GABA_B_Rs. In view of the present results, such benefit could also derive (perhaps principally) from CGP7930 regulating GABA_A_Rs, which are targets for the alleviation of symptoms associated with many of these phenotypes (Mohler, 2006; Rudolph and Knoflach, 2011). Thus, dissociating the contribution of GABA_A_Rs in the CGP7930 *in vivo* effect will be challenging. Indeed, baclofen administration to rodents is noted to have antidepressant effects whereas CGP7930 similarly applied, exhibits antidepressant and anxiolytic profiles (Frankowska et al., 2007). This may be due to allosteric modulation of GABA_A_Rs consistent with the similar neuropharmacological profile of CGP7930 and benzodiazepines on IPSC kinetics (Hajos et al., 2000). In accord with this interpretation, a moderate impact of CGP7930 has been observed by comparison with the benzodiazepine chlordiazepoxide in a range of anxiety tests (Jacobson and Cryan, 2008). Significantly, these tests for anxiety were unaffected by ataxic or hypothermic effects (Jacobson and Cryan, 2008) that typically follow GABA_B_R activation. Furthermore, CGP7930's anxiolytic profile closely follows that of diazepam in the elevated-zero maze test, whilst baclofen is not anxiolytic (Frankowska et al., 2007). Interestingly, CGP7930 is more effective in decreasing cocaine self-administration than the GABA_B_R PAM GS39783 (Smith et al., 2004) despite similar PAM activity in biochemical studies (Urwyler et al., 2005).

In conclusion, GABA_A_Rs are also widely modulated by CGP7930 and many actions of this PAM could be due to modulation, at least in part, to the regulation of this GABA receptor subtype. The discovery that K^+^ channels Kir3.1/3.2 are also direct targets for CGP7930, as well as an outward rectifying channel, further complicates the *in vivo* profile of this ligand, and significantly compromises its use as a specific PAM for GABA_B_ receptors.

## Contributions

Conceptualisation – SBH, Electrophysiology of HEK cells – RP, GK, Electrophysiology of neurons – SBH, Analysis of data – SBH, RP, TGS. Project supervision and funding acquisition – SBH and TGS. Writing, reviewing and editing the manuscript – SBH, RP, TGS.

## Declaration of competing interest

The authors declare that there are no competing interests.

## Data Availability

Data will be made available on request.
